# Bone mineral density in patients using aromatase inhibitors: a clinical, nutritional, and quality of life assessment

**DOI:** 10.1590/1807-3107bor-2025.vol39.023

**Published:** 2025-02-21

**Authors:** Alessandra Figueiredo de SOUZA, Sâmila Gonçalves BARRA, Amanda Leal ROCHA, Larissa Marques BEMQUERER, Sicilia Rezende OLIVEIRA, Larissa Nayane CARVALHO, Tânia Mara Pimenta AMARAL, Claudia Borges BRASILEIRO, Fernando Oliveira COSTA, Leandro Napier SOUZA, Bruno Muzzi CAMARGOS, Enaldo Melo de LIMA, Adaliene Versiani Matos FERREIRA, Joyce Elisa HEREDIA, Marina Chaves de OLIVEIRA, Soraia MACARI, Lucas Guimarães ABREU, Ricardo Alves MESQUITA, Tarcília Aparecida SILVA

**Affiliations:** (a) Universidade Federal de Minas Gerais – UFMG, School of Dentistry, Department of Oral Surgery, Pathology and Clinical Dentistry, Belo Horizonte, MG, Brazil.; (b) Universidade Federal de Minas Gerais – UFMG, School of Dentistry, Belo Horizonte, MG, Brazil.; (c) Hospital Mater Dei, Bone Densitometry Center, Belo Horizonte, MG, Brazil.; (d) Hospital Mater Dei, Integrated Cancer Unit, Belo Horizonte, MG, Brazil.; (e) Universidade Federal de Minas Gerais – UFMG, Nursing School, Department of Nutrition, Belo Horizonte, MG, Brazil.; (f) Universidade Federal de Minas Gerais – UFMG, School of Dentistry, Department of Restorative Dentistry, Belo Horizonte, MG, Brazil.; (g) Universidade Federal de Minas Gerais – UFMG, School of Dentistry, Department of Child’s and Adolescent’s Oral Health, Belo Horizonte, MG, Brazil.

**Keywords:** Aromatase Inhibitors, Estrogens, Osteoporosis, Bone Density, Periodontal Diseases

## Abstract

The use of aromatase inhibitors (AIs) leads to an imbalance in bone remodeling and can cause osteoporosis. This study aimed to identify clinical, periodontal, nutritional, and biochemical determinants of bone mineral density (BMD) changes in patients using AIs. The sample consisted of 40 women using AIs and 32 controls. BMD was assessed by dual X-ray absorptiometry (DXA). Data on nutritional, anthropometric, oral and periodontal status, and oral health-related quality of life (OHRQoL) were collected. Cytokines and adipokines were quantified in saliva and serum. Thirty-nine of the 72 women had low BMD, with a similar distribution in the control and AIs groups. BMD was lower in older women using AIs (p = 0.009) and in smokers (p = 0.034). Anthropometric assessment demonstrated that women with low BMD who used AIs had lower weight (p = 0.028). Although the frequency of periodontitis was similar in all groups, higher IL-6 (p = 0.004), IL-1β (p = 0.002), and IL-33 (p = 0.006) levels were associated with poor periodontal status. Women who used AIs were 1.18 times more likely to report better OHRQoL than controls. While advanced age, smoking, and lower weight are factors associated with low BMD, the use of antiresorptive agents was a protective factor for maintaining BMD in women using AIs.

## Introduction

Breast cancer is the most commonly diagnosed cancer in women, with an estimated incidence of 2.3 million new cases worldwide in 2020.^1^ Aromatase inhibitors (AIs) such as anastrozole, exemestane, and letrozole are the gold standard adjuvant therapy.^2^ These drugs act by inhibiting cytochrome P450 (aromatase), which catalyzes the conversion of androgens to estrogens. They also decrease the proliferation of breast cancer cells by stimulating cell cycle arrest and enhancing apoptosis.^3^ The adverse effects of AIs include hot flashes, arthralgias, myalgias, and an imbalance between bone formation and bone resorption that causes diseases such as osteoporosis.^3,4^

Osteoporosis is a metabolic bone disorder characterized by the microarchitectural deterioration of bone tissue that predisposes individuals to an increased risk of fractures. Independent risk factors for osteoporosis include low bone mineral density (BMD), advanced age, time since menopause, a family history of osteoporosis, smoking, and body mass index (BMI).^5,6^ Moreover, poor dietary habits such as the consumption of high-calorie diets rich in refined carbohydrates and fat and low calcium intake are associated with osteopenia and osteoporosis.^7,8^ The diagnosis of osteoporosis is made by BMD assessment at different skeletal sites, usually femur and lumbar spine, by dual X-ray absorptiometry (DXA).^2^ Moreover, the jaws may exhibit osteoporosis-induced changes that can be useful as an ancillary tool for the diagnosis of low BMD.^9^

Current evidence suggests that systemic bone loss compromises alveolar bone microarchitecture, making it more susceptible to periodontitis, resorption and consequently tooth loss.^10,11^ A possible explanation for this association is that the systemic inflammation associated with osteoporosis increases the inflammatory response in periodontal tissues, favoring alveolar bone loss.^11,12^

Considering the risk of bone loss and involvement of alveolar bone in patients using AIs, the aim of the present study was to assess clinical, periodontal, and inflammatory salivary and blood parameters in these individuals. In addition, recognized risk factors for low BMD such as nutritional and anthropometric data were assessed and oral health-related quality of life (OHRQoL) was evaluated. Our hypothesis was that the use of AIs worsens oral-health outcomes. These data can be useful to identify additional determinants of BMD in patients using AIs.

## Methodology

### Study design, recruitment period, and ethical issues

This cross-sectional study was approved by the Institutional Ethics Committee (Protocol 84967518.0.0000.5149). The Education Board and the Research Committee of Mater Dei Hospital also gave their approval (Protocol 84967518.0.3001.5128). The study was conducted in accordance with the guidelines established in the Declaration of Helsinki (1964).

The case group consisted of women using AIs who underwent treatment at the Integrated Cancer Unit of Mater Dei Hospital. Participants were referred to the Bone Densitometry Service because of the presence of risk factors for osteoporosis. Patients were recruited from August 2018 to December 2019. The control group consisted of women not using AIs who were recruited in the waiting room of the Bone Densitometry Service of Mater Dei Hospital from October 2019 to August 2021. Sociodemographic and medical data, comorbidities, medication use, and blood tests [C-terminal telopeptide of type I collagen, calcium, ionic calcium, vitamin D, estrogen (17β-estradiol, estriol, estrone), glucose, insulin, cholesterol, and triglycerides] were recorded.

**Figure 1:**
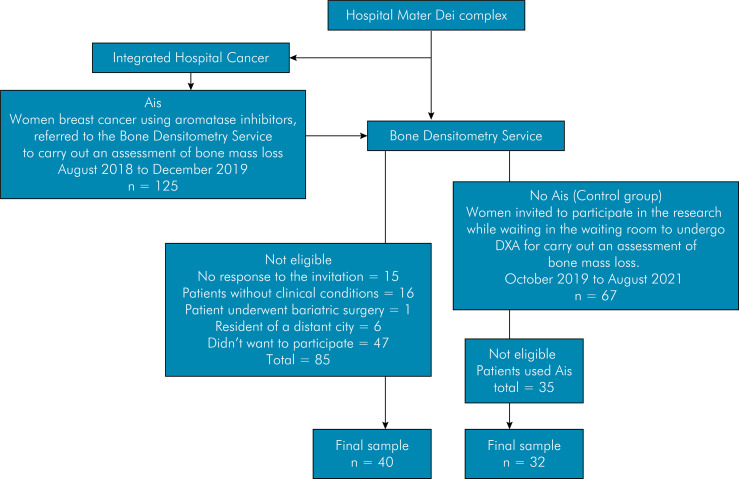
Flowchart of the sampling strategy and reasons for inclusion/exclusion of individuals using or not using aromatase inhibitors (AIs).

**Figure 2:**
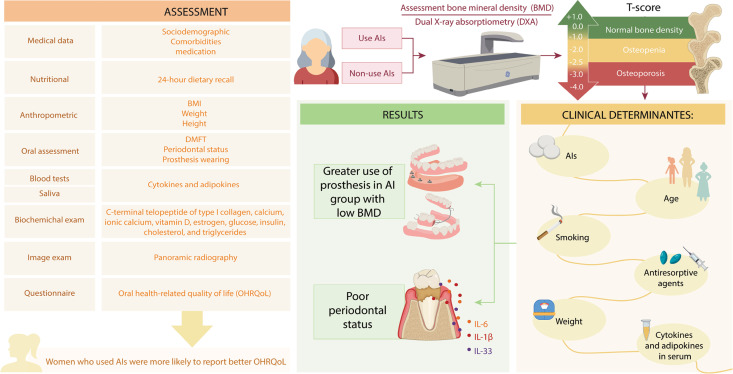
Graphic illustration of the experimental design and main findings of the study. The sample consisted of women using aromatase inhibitors (AIs) and women not using AIs. All participants underwent bone mineral density (BMD) evaluation by dual X-ray absorptiometry (DXA). Based on the T-score parameters obtained from the DXA examination, the patients were categorized as having low bone mass (T-score <-1; osteopenia and osteoporosis) or normal bone density (T-score ≥ -1). Simultaneously, all women underwent an assessment of clinical data (sociodemographic factors, comorbidities, and medication usage); nutritional data (24-hour dietary recall); anthropometric measurements (BMI, weight, and height); oral examination (DMFT: tooth decayed, missing, and filled index, periodontal status, and prosthesis wearing); blood tests and saliva analysis (cytokines and adipokines); biochemical examinations (C-terminal telopeptide of type I collagen, calcium, ionic calcium, vitamin D, estrogen, glucose, insulin, cholesterol, and triglycerides); imaging examination (panoramic radiography), and oral health-related quality of life (OHRQoL). The main clinical determinants for BMD alteration identified in the study were the use of AIs, advanced age, smoking, use of antiresorptive agents, weight, and the presence of adipokines in the serum (leptin and resistin). Both groups of women (using or not AIs) exhibited worse periodontal health status and an increase in cytokines (IL-1β, IL-6, and IL-33 in saliva). Women using AIs and experiencing reduced bone mass exhibited a higher prevalence of prosthesis usage. Interestingly, women who used AIs were more likely to report better OHRQoL.

Individuals who agreed to participate received an informed consent form for reading and signing. Patients younger than 18 years, men, women with fewer than 6 teeth and with a history of head and neck tumors, women who had undergone periodontal treatment in the last 6 months, women who had used antibiotics for up to 3 months before periodontal evaluation, and women whose medical records contained insufficient clinical information were excluded. A flowchart showing the sampling strategy, inclusion/exclusion criteria, and final sample in both groups is presented in Figure 1. Figure 2 provides a graphic presentation of the study’s experimental design and main results.

### Dual-energy X-ray absorptiometry and assessment of bone mineral density

A Lunar iDXA densitometer (GE Healthcare, Chicago, USA) was used for DXA. Exams were performed at the same center and by the same operator. BMD was assessed according to the recommendations of the International Society for Clinical Densitometry.^13^ The regions of the lumbar spine (L1-L4) and proximal femur (neck and total) were examined. The lowest region of interest (ROI) T-score between the neck and total femur was considered for assessment of the proximal femur. The lowest T-score between the lumbar spine and proximal femur was used for the determination of BMD. The absolute BMD values in grams per square centimeter (g/cm^2^) were compared to determine BMD-monitored differences among the examinations of each patient. BMD was calculated using the enCORE program (version 14.1; GE Healthcare). The results are expressed as standard deviations (SD) and are reported as T- or Z-scores. For the calculation of T-scores, the recommended reference range uses femoral neck measurements obtained for Caucasian women aged 20 to 29 years from the National Health and Nutrition Examination Survey (NHANES) III reference database.^14^

Based on the DXA scores and World Health Organization criteria, the participants were allocated into two groups according to the score obtained: (a individuals with normal BMD (T-score ≥ -1) and b) individuals with low BMD including those with osteopenia (-1 > T-score > -2.5) and those with osteoporosis (T-score ≤ -2.5).^15^

### Oral health outcomes and oral health-related behaviors

Oral assessment of each participant consisted of full mouth clinical and imaging examinations. The total decayed, missing and filled teeth (DMFT) index and prosthesis wearing were recorded. Periodontal status was evaluated by one intra-calibrated examiner (AFS) with a periodontal probe PCP15 (Hu-Friedy^®^, Chicago, USA). The following parameters were recorded: plaque index, probing depth (PD), clinical attachment level (CAL), and bleeding on probing (BOP). Six measurements of PD and CAL (circumferential probing) were performed per tooth on all teeth, with the exception of the third molars that were only evaluated when they occupied the position of second molars and were functioning and completely erupted. The highest value for each site (mesial, buccal, distal, and lingual) was recorded, summing four values per tooth. The participants were classified according to the 2018 classification of periodontitis as healthy or stage I, II, III, or IV. In both groups (cases and controls), the participants were further classified into individuals without periodontitis [healthy and stage I (borderline between gingivitis and periodontitis) were grouped for data analysis] and individuals with periodontitis (stage II, III, or IV). Data on oral health-related behaviors (frequency of tooth brushing, time since last visit to the dentist, and reason for visiting the dentist) were also collected.

### Saliva collection and processing

For unstimulated sialometry, all saliva produced in the mouth was collected for 5 minutes into a sterile 50-mL tube. For stimulated sialometry, the participants were asked to chew a hyperboloid for 5 minutes and to spit the whole produced saliva into a 50-mL tube. Salivary flow was determined by dividing the total volume of saliva collected by five (mL/min). The samples were centrifuged at 3,000 rpm for 15 minutes at 4^o^C. The supernatants were collected, diluted (1:1) in phosphate-buffered saline (0.4 mM NaCl and 10 mM NaPO4, pH7.4) containing protease inhibitors (0.1 mM phenylmethylsulfonyl fluoride, 0.1 mM benzethonium chloride, 10 mM ethylenediaminetetraacetic acid, 0.01 mg/mL aprotinin A, and 0.05% Tween-20), and frozen at -80^o^C until analysis of saliva samples for cytokine quantification.^17^

### Blood collection

Peripheral blood samples were collected into tubes containing K2EDTA (BD VacutainerTM, Franklin Lakes, USA). Samples were centrifuged at 450 g for 10 min at room temperature in order to obtain plasma. Blood samples were used for cytokine and adipokine quantification^17^.

### Cytokine and adipokine quantification

Cytokines [tumor necrosis factor-alpha (TNF-α) and interleukin (IL)-1β, IL-6, IL-8, IL-10, and IL-12] were measured in unstimulated saliva using the CBA Human Inflammatory Cytokine Kit (BD Biosciences, San Diego, USA). The samples were read in a flow cytometer (BD FACSVerse Flow Cytometer, Becton Dickinson, San Jose, USA). Concentrations of IL-33, osteoprotegerin (OPG) and receptor activator of nuclear factor kappa-Β ligand (RANKL) were also measured in serum and unstimulated saliva. Adipokines (leptin, resistin, and adiponectin) and osteocalcin were quantified only in serum. Assays were done by sandwich ELISA using commercial kits (DuoSet^®^ ELISA, R&D Systems, Minneapolis, USA). The concentrations of cytokines and adipokines were measured using a standard curve according to the manufacturer’s guidelines. The results were expressed as picogram of cytokine/adipokine per mL.

### Nutritional and anthropometric assessment

A 24-hours dietary recall that recorded all foods consumed on the previous day was used. Based on these data, the total energy value of the diet (kcal), macronutrients (carbohydrates, proteins, and lipids), some micronutrients (magnesium, phosphorus, potassium, vitamin D, and calcium), and some food groups (dairy products, animal protein, fruits and vegetables, flour, pasta, and bread) were determined using the Dietbox software and the table of the nutritional composition of foods consumed in Brazil^18^. The vitamin D and calcium values were also included the supplementation values.

Weight was measured on a platform weighing scale (Model 680 Family, Tanita Corporation). Height was measured with a stadiometer to the nearest 0.1 cm (Model EST-221, Balmak). Waist circumference, abdominal circumference, and hip circumference were obtained with a measuring tape. The BMI [weight (kg)/height^2^ (m)] and waist/hip ratio were also calculated^19^.

### Oral health-related quality of life

The OHRQoL questionnaire validated for Brazilian Portuguese consisted of 16 key questions distributed across three subscales: physical, social, and psychological. The total score ranges from 16 (worst quality of life) to 80 (best quality of life). The response scale was as follows: “very bad” (score 1), “bad” (score 2), “none” (score 3), “good” (score 4), and “very good” (score 5)^20^.

### Statistical analysis

Statistical analysis was performed using the Statistical Package for the Social Sciences (version 24.0; SPSS, IBM Incorp., Armonk, USA). Chi-square tests (Fisher’s exact and Pearson’s tests) and the Student *t-*test were used for intergroup comparisons. Intergroup comparisons between the AIs and non-AIs groups were performed. In both the AI and non-AI groups, intragroup comparisons between individuals with low BMD and those with normal BMD were also performed. Intergroup and intragroup comparisons were performed for sociodemographic characteristics, medical conditions, laboratory tests, oral health outcomes, sialometry, oral health-related behaviors, saliva cytokines, blood cytokines, and anthropometric parameters.

The Student *t-*test was used to compare oral health outcomes, sialometry, saliva cytokines, and blood cytokines between women with and without periodontitis and multivariate analysis was performed to identify the factors associated with periodontitis.

Intragroup (AIs X non-AIs) and intergroup (low BMD X normal BMD) comparisons of the subscale scores and total score of the OHRQoL questionnaire were performed using the Mann-Whitney test. Poisson regression was used to compare the total OHRQoL score between women who used AIs and those who did not, controlling for confounding variables. The rationale for choosing the confounding variables was p < 0.20 in the bivariate analysis. The level of statistical significance was set at p < 0.05 for all analyses.

## Results

### Demographic and clinical variables

Seventy-two women were evaluated in this study; of these, 40 (56%) used AIs [24 (60%) letrozole, 14 (35%) anastrozole, and 2 (5%) exemestane] and 32 (44%) did not use AIs. Thirty-nine (57.4%) of the 72 women had low BMD, 21 (52.5%) in the AIs group and 18 (64.3%) in the control group.

Table 1 shows the comparison of demographic and clinical variables between women who used AIs and those who did not use AIs according to BMD. The age of patients was similar in both groups (p = 0.545). However, in the AIs group, patients with normal BMD were younger than those with low BMD (p = 0.009). There was a significant difference in smoking (p = 0.034) and smoking duration (p = 0.012) between AI groups, with higher values in those with low BMD. In the control group, women with low BMD also had a longer smoking duration (p = 0.014) (Table 1).

**Table 1:** Demographic and clinical findings of individuals using or not using aromatase inhibitors (AIs) according to bone mineral density (BMD) status.

Variables	No Ais (n = 28)			Ais (n = 40)			No AIs x AIs
	Normal BMD (n=10)	Low BMD (n=18)	p-value	Normal BMD (n=19)	Low BMD (n=21)	p-value	p-value
	n (%)	n (%)		n (%)	n (%)		
Age (years^)a^	56.90 (± 7.78)	62.72 (± 8.00)	0.074*	56.95 (± 11.14)	65.90 (± 9.49)	0.009*	0.545*
Age at menarche (years)^a^	11.60 (± 1.07)	12.44 (± 1.88)	0.206*	12.47 (± 1.71)	13.05 (± 1.82)	0.316*	0.328*
Oophorectomy							
Yes	2 (20.00)	3 (16.70)	1.000***	4 (21.10)	4 (19.00)	1.000***	0.762**
No	8 (80.00)	15 (83.30)		15 (78.90)	17 (81.00)		
Oophorectomy duration^a^	21.50 (± 20.50)	23.33 (± 19.60)	0.926*	17.00 (± 7.21)	15.00 (± 12.76)	0.825*	0.439*
Age at oophorectomy (years)^a^	35.50 (± 14.84)	43.00 (± 19.15)	0.676*	46.00 (± 3.00)	49.67 (± 4.61)	0.313*	0.340*
Hysterectomy							
Yes	5 (50.00)	7 (38.90)	0.698***	8 (42.10)	11 (52.40)	0.545**	0.637**
No	5 (50.00)	11 (61.10)		11 (57.90)	10 (47.60)		
Hysterectomy duration^a^	20.00 (± 15.23)	33.29 (± 6.15)	0,061*	23.86 (± 11.23)	29.44 (± 12.07)	0.360*	0.835**
Menopause							
Yes	8 (80.00)	18 (100.00)	0.119***	18 (94.70)	(21) 100.00	0.475***	0.317***
No	2 (20.00)	0 (0.00)		1 (15.30)	0 (0.00)		
Menopause duration^a^	9.86 (± 5.30)	13.67 (± 8.79)	0.298*	12.13 (± 8.22)	16.00 (± 10.17)	0.226*	0.375*
Age at menopause, (years)^a^	48.57 (± 3.82)	49.06 (± 6.21)	0.850*	45.63 (± 6.28)	48.95 (± 5.29)	0.094*	0.259*
Race							
White	7 (70.00)	14 (77.80)		13 (68.40)	14 (66.70)		
Black	1 (10.00)	2 (11.10)	0.814***	5 (26.30)	3 (9.50)	0.180***	0.668**
Mixed	2 (10.00)	2 (11.10)		1 (5.30)	5 (23.80)		
Education							
Primary	2 (20.00)	2 (11.10)		3 (15.80)	3 (14.30)		
High school	2 (20.00)	8 (44.4)	0.479***	8 (42.10)	10 (47.60)	1.000***	0.855**
Graduate	6 (60.00)	8 (44.4)		8 (42.10)	8 (38.10)		
Smoking							
Yes	2 (20.00)	10 (55.60)	0.114***	2 (10.50)	9 (42.90)	0.034**	0.213**
No	8 (80.00)	8 (44.40)		17 (89.50)	12 (57.10)		
Smoking duration (years)^a^	3.50 (± 7.47)	16.94 (± 19.03)	0.014*	2.63 (± 8.55)	15.24 (± 19.67)	0.012*	0.505*
Alcohol intake							
Yes	4 (40.00)	11 (61.10)	0.433***	7 (36.80)	12 (57.10)	0.225**	0.813**
No	6 (60.00)	7 (38.90)		12 (63.20)	9 (42.90)		
Physical activity							
Yes	4 (40.00)	8 (44.40)	1.00***	9 (47.40)	12 (57.10)	0.752**	0.240**
No	6 (60.00)	10 (55.60)		10 (52.60)	9 (42.90)		
History of falls							
Yes	4 (40.00)	12 (66.70)	0.243***	8 (42.10)	8 (38.10)	1.000**	0.343**
No	6 (60.00)	6 (33.30)		11 (57.90)	13 (61.90)		
Fracture							
Yes	2 (20.00)	1 (16.70)	1.000***	1 (5.30)	3 (14.30)	0.607***	0.323***
No	8 (80.00)	15 (83.30)		18 (94.70)	18 (85.70)		
Antiresorptive							
Yes	0 (0.00)	8 (33.30)	0.062***	5 (26.30)	14 (66.70)	0.014**	0.013**
No	10 (100.00)	12 (66.70)		14 (73.70)	7 (33.30)		
Antiresorptive duration (months)^a^	0.00 (± 0.00)	22.33 (± 41.27)	0.035*	11.58 (± 30.01)	36.48 (± 53.11)	0.080*	0.206*
AIs → Yes (duration months)^a^	-	-	-	23.32 (± 17.60)	30.43 (± 19.64)	0.237*	**-**
Bisphosphonate n (%)							
Yes	0 (0.00)	6 (33.30)	0.062***	5 (26.30)	11 (52.40)	0.117**	0.072**
No	10 (100.00)	12 (66.70)		14 (73.70)	10 (47.60)		
Denosumab							
Yes	0 (0.00)	1 (5.60)	1.000***	3 (15.80)	6 (28.60)	0.457***	0.035***
No	10 (100.00)	17 (94.40)		16 (84.20)	15 (71.40)		

^a^ Values represent the mean of the groups with and without bone loss; *Student’s t test; **Pearson’s test; ***Fisher’s exact test. (±) = Standard deviation.

Diagnosis of comorbidities (systemic arterial hypertension, coronary artery disease, diabetes mellitus, depression, dyslipidemia, thyroid, organ transplant, chronic renal failure and others) were compared between groups. In the control group, the number of women with hypothyroidism was significantly higher in the subgroup with normal BMD (p = 0.035). The analysis of other comorbidities showed similar rates for all variables (data not shown).

### Antiresorptive therapy, adjuvant cancer treatments and other medications

The use of antiresorptive agents was significantly higher among women in the AIs group with low BMD (p = 0.014). The use of this medication was also higher in the AIs group compared to control (p = 0.013). In the control group, the duration of antiresorptive therapy was longer in women with low BMD (p = 0.035). The use of denosumab was also higher in the AIs group (p = 0.035) (Table 1).

Among women taking AIs, antiresorptive therapy with bisphosphonates was used by 40%, zoledronic acid by 27.5%, risedronic acid by 5%, alendronic acid by 5%, and zoledronic acid/alendronic acid by 2.5%. In the control group, 18.7% of the women used bisphosphonates, 9.4% alendronic acid, 6.3% risedronic acid, and 3.1% ibandronic acid. Overall, no differences in bisphosphonates therapy were observed (Table 1).

In the AIs group, adjuvant cancer treatments included chemotherapy (52.5%) and radiotherapy (92.5%). Regarding other medications used, statins were more frequently used in the AIs group (p = 0.007). Calcium supplements were also more frequently used by women in the AIs group with low BMD (p = 0.010). In the control group, the use of steroids was significantly higher among women with normal BMD (p = 0.037). No differences in the other medications were observed between groups (data not shown).

### Oral health outcomes and oral health-related behaviors

The frequency of medication-related osteonecrosis of the jaws (MRONJ) was similar between groups, with two cases observed in the AIs group and one case in the control group (Table 2).

**Table 2:** Oral health outcomes and oral health-related behaviors of individuals using or not using aromatase inhibitors (AIs) according to bone mineral density status (BMD).

Variables	No Ais (n = 28)			Ais (n = 40)			No AIs x AIs
	Normal BMD^a^ (n = 10)	Low BMD^b^ (n = 18)	p-value	Normal BMD^a^ (n = 19)	Low BMD^b^ (n = 21)	p-value	p-value
	n	n		n	n		
Decayed teeth	0.30	0.17	0.508*	0.32	0.14	0.385*	0.353*
Missing teeth	5.20	8.06	0.275*	5.74	8.19	0.329*	0.997*
Filled teeth	12.60	12.83	0.913*	13.84	14.14	0.892*	0.435*
Sound teeth	9.90	6.94	0.125*	8.11	5.52	0.125*	0.424*
Number of teeth	22.60	19.94	0.315*	22.26	19.81	0.329*	0.968*
DMFT	18.10	21.06	0.125*	19.84	22.48	0.117*	0.436*
PD (mm)	1.98	2.04	0.738*	1.91	1.88	0.842*	0.128*
CAL (mm)	2.13	2.37	0.295*	2.21	2.26	0.730*	0.608*
BOP (%)	6.90	8.82	0.578*	7.61	6.95	0.730*	0.370*
PI (%)	24.40	17.76	0.430*	11.94	10.05	0.718*	0.035*
Periodontitis, n (%)							
Healthy and stage I	3 (30.00)	5 (29.40)	1.000***	5 (27.80)	3 (15.80)	0.447***	0.777**
Stage II, III or IV	7 (70.00)	12 (70.60)		13 (72.20)	16 (84.20)		
Sialometry (unstimulated), mean (mL/min)	2.480	2.656	0.854*	0.763	0.681	0.607*	0.512*
Sialometry (stimulated), mean (mL/min)	6.210	7.200	0.709*	1.979	1.741	0.433*	0.599*
Osteonecrosis, n (%)							
Yes	0 (0.00)	1 (5.60)	1.000***	1 (5.30)	1 (4.80)	1.000***	1.000***
No	10 (100.00)	17 (94.40)		18 (94.70)	20 (95.20)		
Prothesis wearing, n (%)							
Yes	4 (40.00)	7 (38.90)	1.000***	3 (15.80)	11 (52.40)	0.022**	0.630*
No	6 (60.00)	11 (61.10)		16 (84.20)	10 (47.60)		
Tooth brushing (times/day), n (%)							
1 times/day	0 (0.00)	0 (0.00)		0 (0.00)	1 (4.80)		
2 times/day	2 (20.00)	8 (44.40)	0.247***	3 (17.50)	3 (14.30)	1.000***	0.055***
> 2 times/day	8 (80.00)	10 (55.60)		14 (82.40)	17 (81.00)		
When was your last visit to the dentist? n (%)							
< 1 years	9 (90.00)	16 (88.90)		15 (78.90)	16 (76.20)		
> 1 < 3 years	1 (10.00)	2 (11.10)	1.000***	2 (10.50)	4 (19.00)	0.741***	0.247***
> 3 years	0 (0.00)	0 (0.00)		2 (10.50)	4 (19.00)		
What was your reason for visiting the dentist? n (%)							
Check-up	3 (30.00)	6 (33.30)		9 (47.40)	7 (35.00)		
Treatment	5 (50.00)	8 (44.40)	1.000***	8 (42.10)	13 (65.00)	0.176***	0.054**
Pain / emergency	2 (20.00)	4 (22.20)		2 (10.50)	0 (0.00)		

^a^Values representing the subgroup means without low BMD; ^b^Values representing the means of the subgroup with low BMD; *Student’s t test; **Pearson’s test; ***Fisher’s exact test. DMFT: Tooth. Decayed. Missing and Filled Index; PD: Probing depth; CAL: Clinical attachment Level; BOP: Bleeding on probing; PI: Plaque index.

In the AIs group, the proportion of participants who wore a prosthesis was significantly higher among women with low BMD compared to those with normal BMD (p = 0.022). The plaque index of controls was significantly higher than that of women who used AIs (p = 0.035). The groups were similar for DMFT index (p = 0.436), PD (p = 0.128), CAL (p = 0.608), BOP (p = 0.370), and diagnosis of periodontitis (p = 0.777). However, the number of patients with stage II, III or IV periodontitis tended to be higher in the AIs group. The proportion of participants with stage II, III or IV periodontitis was also higher among women with low BMD compared to those with normal BMD (Table 2).

### Serum and salivary analysis

The unstimulated (p = 0.512) and stimulated (p = 0.599) salivary flow rates were similar in women who used AIs and controls. No differences in the other oral health behaviors were observed between groups (p > 0.05) (Table 2).

Table 3 displays the comparisons of saliva and blood cytokines. In the control group, the salivary levels of RANKL (p = 0.039) and IL-33 (p = 0.006) were significantly higher among women with low BMD compared to those with normal BMD. In the AIs group, no differences in RANKL or IL-33 levels were observed according to BMD (p = 0.253 and p = 0.582, respectively). The blood concentrations of resistin (p < 0.001) and leptin (p < 0.001) were significantly higher in controls compared to the AIs group (Table 3). There were no differences in the laboratory findings between groups or subgroups (data not shown).

**Table 3:** Concentration of cytokines in saliva and blood of individuals using or not using aromatase inhibitors (AIs) according to bone mineral density status (BMD).

Cytokines (pg/mL)	No Ais (n = 28)			Ais (n = 40)			No AIs x Ais
	Normal BMD^a^ (n = 10)	Low BMD^b^ (n = 18)	p-value	Normal BMD^a^ (n = 19)	Low BMD^b^ (n = 21)	p-value	p-value
	n (±)	n (±)		n (±)	n (±)		
Saliva							
RANKL	61.61 (± 35.66)	106.36 (± 56.05)	0.039*	91.54 (± 60.20)	71.51 (± 48.71)	0.253*	0.187*
OPG	783.22 (± 416.87)	1025.77 (± 1042.78)	0.511*	595.13 (± 334.59)	644.35 (± 353.59)	0.655*	0.062*
IL 33	0.94 (± 2.84)	12.59 (± 15.45)	0.006*	24.56 (± 53.59)	17.11 (± 28.69)	0.582*	0.207*
IL1 - β	63.51 (± 26.56)	103.90 (± 84.35)	0.328*	81.19 (± 74.03)	71.64 (± 53.06)	0.645*	0.564*
IL6	7.77 (± 4.88)	5.42 (± 4.42)	0.389*	9.85 (± 16.88)	11.00 (± 14.90)	0.823*	0.358*
IL8	876.33 (± 672.77)	705.98 (± 265.78)	0.527*	6290.55(± 22883.51)	2383.27 (± 4987.19)	0.461*	0.442*
IL10	0.40 (± 0.54)	0.00 (± 0.00)	0.178*	0.13 (± 0.41)	0.40 (± 0.50)	0.081*	0.424*
IL12	0.24 (± 0.33)	0.36 (± 0.61)	0.690*	0.46 (± 0.62)	0.41 (± 0.62)	0.789*	0.541*
TNF - α	2.33 (± 0.98)	2.59 (± 1.63)	0.751*	2.42 (± 1.81)	2.34 (± 1.59)	0.880*	0.830*
RANKL	429.16 (± 548.93)	72.67 (± 119.76)	0.063*	122.67 (± 314.35)	113.576 (± 269.30)	0.922*	0.346*
Blood							
OPG	1467.24 (± 316.63)	1243.76 (± 471.62)	0.323*	1051.31 (± 373.76)	1174.985 (± 628.29)	0.460*	0.155*
IL 33	19.20(± 21.77)	9.82 (± 17.60)	0.360*	20.03 (± 34.99)	37.05 (± 87.12)	0.432*	0.367*
Osteocalcin	2424.01 (± 3759.41)	521.03 (± 667.85)	0.133*	57.33 (± 249.90)	94.32 (± 242.29)	0.637*	0.076*
Resistin	8710.99 (± 353.19)	9010.42 (± 351.63)	0.122*	8125.57 (± 1253.22)	7526.92 (± 1842.24)	0.242*	<0.001*
Leptin	5037.99 (± 392.82)	4801.28 (± 1159.77)	0.565*	3099.83 (± 1516.43)	3850.71 (± 1253.13)	0.095*	<0.001*
Adiponectin	15006.91 (± 475.51)	14871.61 (± 496.68)	0.601*	14653.18 (± 785.84)	14797.91 (± 792.22)	0.566*	0.362*

^a^Values representing the subgroup means without low BMD; ^b^Values representing the means of the subgroup with low BMD; *Student’s t test. IL: Interleukin; TNF: Tumor necrosis factor; OPG: osteoprotegerin; RANKL: receptor activator of nuclear factor-kappa B ligand; (±) = Standard deviation.

### Anthropometric and nutritional findings

Comparisons of the anthropometric parameters in the control group showed that women with normal BMD had a higher weight (p = 0.011), height (p = 0.009), and hip circumference (p = 0.020) than those with low BMD. In the AIs group, women with normal BMD exhibited a higher weight (p = 0.028) than those with low BMD. No further differences were observed between groups (Table 4).

**Table 4:** Anthropometric variables of individuals using or not using aromatase inhibitors (AIs) according to bone mineral density status (BMD).

Variables	No Ais (n = 28)			Ais (n = 40)			No AIs x Ais
	Normal BMD^a^ (n = 10)	Low BMD^b^ (n = 18)	p-value	Normal BMD^a^ (n = 19)	Low BMD^b^ (n = 21)	p-value	p-value
	n (±)	n (±)		n (±)	n (±)		
Weight	79.62 (± 12.67)	67.15 (± 10.85)	0.011*	75.82 (± 16.26)	65.91 (± 10.94)	0.028*	0.773*
Height	162.10 (± 6.11)	156.73 (± 4.02)	0.009*	161.92 (± 6.82)	157.91 (± 8.31)	0.106*	0.471*
Body mass index (BMI)	30.33 (± 4.63)	27.42 (± 4.87)	0.135*	28.95 (± 6.06)	26.41 (± 3.93)	0.120*	0.501*
Abdominal circumference	105.38 (± 10.48)	96.20 (± 10.53)	0.113*	97.27 (± 11.53)	92.23 (± 9.26)	0.174*	0.239*
Waist circumference	91.36 (± 6.99)	86.30 (± 11.11)	0.336*	87.30 (± 12.81)	82.57 (± 9.30)	0.229*	0.552*
Hip circumference	116.00 (± 9.54)	104.65 (± 7.61)	0.020*	107.67 (± 9.55)	102.50 (± 7.92)	0.099*	0.280*
Hip waist ratio	0.79 (± 0.06)	0.82 (± 0.07)	0.368*	0.81 (± 0.07)	0.80 (± 0.05)	0.813*	0.831*

^a^Values representing the subgroup means without low BMD; ^b^Values representing the means of the subgroup with low BMD; *Student’s t test. (±) = Standard deviation.

The results of the analysis of food intake showed no differences between groups or subgroups, except for the consumption of lipids (p = 0.027), fiber (p = 0.021), total sugar (p = 0.021), and total energy value (p = 0.034) that were higher among women who used AIs compared to the control group, regardless of BMD status (data not shown).

### Multivariate analysis

Multivariate analysis was also performed to identify the factors associated with periodontitis. Analysis of the association between mean salivary inflammatory cytokine levels and severity of periodontal disease showed higher levels of IL-6 (p = 0.004), IL-1β (p = 0.002), and IL-33 (p = 0.006) in the group with stage II, III or IV periodontitis compared to the group with healthy and stage I periodontitis (data not shown).

### OHRQoL

Evaluation of OHRQoL revealed higher scores (better OHRQoL) of the physical (p = 0.001), social (p = 0.042), and psychological (p = 0.020) subscales, as well as a higher total score (p = 0.005) in women who used AIs compared to those who did not use AIs (Table 5).

**Table 5:** Oral health-related quality of life of individuals using or not using aromatase inhibitors (AIs) according to bone mineral density status (BMD).

Variables	No Ais (n = 28)			Ais (n = 40)			No AIs x Ais
	Normal BMD (n = 10)	Low BMD (n = 18)	p-value	Normal BMD (n = 19)	Low BMD (n = 21)	p-value	p-value
	n (±)	n (±)		n (±)	n (±)		
Physical	22.00 (± 6.12)	17.44 (± 7.31)	0.114*	24.06 (± 5.37)	24.43 (± 4.28)	0.815*	0.001*
Social	18.60 (± 4.37)	16.75 (± 5.71)	0.391*	19.47 (± 4.71)	19.05 (± 5.05)	0.793*	0.042*
Psychological	18.20 (± 4.78)	16.31 (± 5.05)	0.354*	19.29 (± 5.08)	19.19 (± 4.42)	0.947*	0.020*
Total score	58.80 (± 14.83)	50.50 (± 17.02)	0.217*	62.82 (± 14.33)	62.67 (± 13.16)	0.972*	0.005*

^a^Values representing the subgroup means without AI; ^b^Values representing the means of the subgroup with AI; *Mann Whitney. Significance level < 0.05. (±) = Standard deviation.

The Poisson regression model for quality of life explored the combined effect of sociodemographic and clinical factors (self-reported oral health status) on OHRQoL. Regardless of the confounding variables, women who used AIs were 1.18 times more likely to report better OHRQoL than those who did not use AIs (OR = 1.18; CI = 1.05 – 1.33; p = 0.003). This model also showed the following results: women who completed high school (OR = 1.17; CI = 1.05 – 1.33; p = 0.005) and those with a graduate degree (OR = 1.26; CI = 1.04 – 1.51; p = 0.020) were more likely to report better OHRQoL than those with primary education; women who brushed their teeth 2 times/day (OR = 1.33; CI = 1.08 – 1.63; p = 0.007) or >2 times/day (OR =1.33; CI = 1.12 – 1.59; p = 0.001) were more likely to report better OHRQoL than those who brushed their teeth 1 time/day; women who visited the dentist for check-up were more likely to report better OHRQoL than those who visited the dentist for an emergency appointment to relieve pain (OR = 1.32; CI = 1.09 – 1.61; p = 0.004) (Table 6).

**Table 6:** Regression model evaluating factors associated with quality of life between women using or not using aromatase inhibitors (AIs).

Variable	Odds ratio (OR)	95%CI	p-value
Patient group			
Ais	1.18	1.05-1.33	0.003
No Ais	1		
Schooling			
Primary	1		
High school	1.17	1.05-1.33	0.005
Graduate	1.26	1.04-1.51	0.020
Tooth brushing (times/day)			
1 times/day	1		
2 times/day	1.33	1.08-1.63	0.007
> 2 times/day	1.33	1.12-1.59	0.001
What is your reason for visiting the dentist?			
Check-up	1.32	1.09-1.61	0.004
Treatment	1.12	0.91-1.38	0.255
Pain / emergency	1		

## Discussion

The main findings of this study can be summarized as follows: 1) women using AIs, women older than 65 years, and smokers had lower BMD; 2) the use of antiresorptive agents had a protective effect on BMD of AIs users; 3) women using AIs had higher energy intake and those with low BMD also had a lower body weight; 4) women using AIs had lower levels of adipokines (resistin and leptin), regardless of BMD status; 5) the severity of periodontitis was associated with salivary inflammatory cytokines IL-1β, IL-6, and IL-33 but not with AI use or BMD status; 6) women using AIs reported better OHRQoL.

Postmenopausal low BMD, related to estrogen deficiency, is the main contributor to osteoporosis^5^. In postmenopausal women with breast cancer, treatment with AIs causes a relatively rapid decrease in the concentrations of circulating estrogens and increases bone resorption and fracture risk.^3^ In our sample of women using AIs, the factors that contributed to low BMD were age and smoking. The WHO and the National Osteoporosis Foundation (NOF) have indicated aging and smoking as important risk factors for low BMD.^21^ Advanced age is usually associated with a progressive decline in ovarian production of estrogen, with a negative effect on bone formation.^5^ Smoking may affect bone metabolism through indirect pathways, interfering with weight, hormone levels, and oxidative stress.^22^ The harmful effect of cigarette smoking on bone metabolism was demonstrated in our study and has also been widely explored in the literature,^23^ with the damage being cumulative^24^ and associated with long-term tobacco use.^25^

Healthy habits are generally essential for bone metabolism homeostasis.^5,8^ Within this context, assessment of dietary habits, laboratory tests, and anthropometric analysis were conducted to identify biochemical and nutritional parameters that are potentially associated with low BMD in patients using AIs. We found that women using AIs had higher energy intake, and those with low BMD also had a lower body weight. There are extensive studies on the association between weight and bone metabolism and on how poor dietary habits could affect the development of osteoporosis.^26^ According to the literature, body fat may affect bone metabolism through bidirectional mechanisms. Similar to our findings, a previous study found that thinner postmenopausal women are at increased risk of developing osteoporosis due to the reduction in available nutrients, malnutrition, and low levels of estrogen.^27^ On the other hand, obesity and overweight may also be associated with low BMD due to the meta-inflammation related to overweight.^28^

Current nutritional recommendations for individuals using AIs encourage a predominantly plan*t-*based diet that is low in fat and rich in fruits, vegetables, and whole grains.^29^ In our study, lipid and total sugar intake were significantly higher in the AIs group. Interestingly, estrogen affects dietary intake through appetite modulation,^30^ which may have contributed to the amount of total energy consumed by women in the AIs group. However, there is little information on how estrogen suppression affects components of energy balance in patients with breast cancer or whether alterations in energy balance occur independently of previous chemotherapy or radiotherapy.^30^ Within this context, women in the AIs group had lower levels of obesity-related blood adipokines (resistin and leptin) compared to the control group, in agreement with the lower weight observed in this group. These results suggest a higher energy demand in individuals using AIs and may explain their lower weight. Alterations in body composition during chemotherapy, radiotherapy, and surgery or long-term estrogen suppression are mechanisms suggested to contribute to changes in energy balance in AIs users.^31^

Periodontitis is characterized by microbial-associated, hos*t-*mediated inflammation that results in the loss of periodontal attachment.^16^ The condition is associated with an increase in proinflammatory cytokines.^32^ In our study, higher levels of IL-1β, IL-6, and IL-33 were observed in women with poor periodontal status, independent of BMD status. Periodontitis is regulated by mechanisms similar to those found in osteoporosis. Both conditions are characterized by progressive bone loss and share several risk factors, including low estrogen levels.^12^ Although estrogen levels were reduced in our sample, they were not linked to systemic or alveolar bone loss.

AIs can have a negative impact on oral health. Previous studies have reported that AIs worsen periodontal condition, including increased CAL, PD, and alveolar bone loss within the first 18 months of use.^33^ In the present study, no differences in the frequency of periodontitis were seen between AI users and non-users. Some factors may have contributed to this finding. The plaque index was lower in the AIs group and these individuals also had better oral hygiene habits, including a higher frequency of tooth brushing and shorter intervals between visits to the dentist. Similarly, Taichman et al.^34^ did not find a significant impact of AIs use on periodontal health. The authors also attributed the results to the good oral habits of this population.^34^ In addition, we cannot rule out that other variables might exert a potential protective effect on periodontitis, such as the use of antiresorptive agents^35^ and statins^36^ and supplementation with calcium.^37^

Antiresorptive agents are usually given to pre- and postmenopausal women at the beginning of AIs therapy to prevent bone loss.^21,38^ Indeed, the use of antiresorptive agents was a protective factor against bone loss in the present sample. Many potential mechanisms have been proposed to explain the adjuvant activity of antiresorptive agents.^39^ Clinical trials of bisphosphonate and denosumab use in postmenopausal women with early-stage breast cancer who used AIs have demonstrated a positive impact on BMD.^40^ Despite the widespread use of antiresorptive agents in our sample, interestingly, only three cases of MRONJ lesions were observed. Two patients belonged to the AI group, one using risedronate (for 18 months) and the other using zoledronic acid (for 84 months) followed by denosumab (for 18 months). The lesion identified in the control group was associated with the use of risedronate for 24 months.

Potential limitations of the present study were the relatively small sample and that participants from a private service were originally enrolled, where AIs users had better access to dental care. Dental evaluation and treatment prior to antineoplastic and antiresorptive therapy are routine procedures in private cancer centers.^41^ These factors may also explain the better OHRQoL of women using AIs. On the other hand, the strengths of this study include the use of a well-standardized and comprehensive criteria for BMD determination combined with the wide-ranging analysis of clinical, demographic, saliva, blood, nutritional, and anthropometric parameters.

## Conclusion

Antiresorptive agents are widely prescribed because of the potential deleterious effects of AIs on bone; however, complications such as MRONJ were rare in our cohort. Periodontitis was associated with salivary levels of IL-6, IL-1β, and IL-33 but not with AIs use or BMD status. While age, smoking, and low weight were found to be significantly correlated with low BMD, the contribution of supplementary nutrition and anthropometric data, cytokines, and adipokines requires further investigation. Overall longitudinal analysis of these factors, including detailed clinical and laboratory examination at baseline, is necessary to hierarchize the determinants of BMD status in individuals using AIs and to provide personalized medical and dental care for this group.
